# Immunophenotype classification and therapeutic outcomes of Chinese primary gastrointestinal diffuse large B-cell lymphoma

**DOI:** 10.1186/1471-230X-12-77

**Published:** 2012-06-25

**Authors:** Zizhen Zhang, Yanying Shen, Danping Shen, Xingzhi Ni

**Affiliations:** 1 Department of General Surgery, Ren Ji Hospital Medical College of Shanghai Jiao Tong University, Dongfang Road 1630, Shanghai, 200127, China; 2 Department of Pathology, Ren Ji Hospital, Medical College of Shanghai Jiao Tong University, Dongfang Road 1630, Shanghai, 200127, China

**Keywords:** Diffuse large B-cell lymphoma, Gastrointestinal lymphoma, Immunophenotype, Chemotherapy, Prognosis

## Abstract

****Background**:**

Recent studies showed that diffuse large B-cell lymphoma (DLBCL) could be classified into germinal centre B cell-like (GCB) and non-germinal centre B cell-like (non-GCB) phenotypes according to CD10,Bcl-6 and MUM1 expression. But primary gastrointestinal DLBCL has rarely been studied. This study was aimed to investigate the relationship between immunophenotypic classification, therapeutic outcomes and the prognosis of patients with primary gastrointestinal DLBCL.

****Methods**:**

Between 1998 and 2010, there were 151 patients studied at Shanghai Renji Hospital with a histopathological diagnosis of primary gastrointestinal DLBCL. Immunohistochemistry was performed using EnVision methods for CD10, BCL-6 and MUM1. The clinicopathologic features and follow-up data were analyzed by the Kaplan-Meier method, log-rank test and χ2 test.

****Results**:**

According to the expression of CD10, BCL-6 and MUM1, 31.8 % (48/151) of the cases belonged to the GCB subtype and 68.2 % (103/151) belonged to the non-GCB subtype. There was a significant difference of local lymph node metastasis between the GCB and non-GCB groups (P < 0.05). Patients in the GCB group had a better survival rate than those in the non-GCB group (5-year survival rate, 65.2 % vs 36.4 %, P < 0.05). In the GCB group, there was no significant difference in survival rates in patients receiving R-CHOP and CHOP therapy (P > 0.05). In the non-GCB group, the survival rate in patients treated with R-CHOP therapy was significantly longer than those treated with CHOP therapy (5-year survival rate, 62.8 % vs 30.8 %, P < 0.05).

****Conclusions**:**

The immunophenotype classification of gastrointestinal DLBCL, which is closely related to local lymph node metastasis, is found to have prognostic significance. Immunophenotype classification is also useful in selecting the chemotherapy protocol.

## **Background**

In 2004, Hans et al found that according to the expression of CD10, BCL-6 and MUM-1, diffuse large B-cell lymphoma (DLBCL) could be categorized into two subtypes: germinal centre B cell-like (GCB) and non-germinal centre B cell-like (non-GCB) phenotypes. This immunophenotype classification could be used to predict the survival rate in patients with DLBCL [1]. However, primary gastrointestinal DLBCL has rarely been studied with the immunophenotype classification.

The gastrointestinal tract is the most common extranodal site for lymphoma and represents 30-40 % of all extranodal sites. All histopathological categories of nodal lymphomas may also arise in the gastrointestinal tract, but DLBCL is the most common histopathological subtype, which accounts for about 50 %. However, there is still debate on the diagnosis, treatment and prognosis of gastrointestinal DLBCL. Thus, the main goals of the current study were: (1) to evaluate if immunohistochemial staining with antibodies of CD10, BCL-6 and MUM1 could define distinct, clinically significant subgroups of gastrointestinal DLBCL; (2) to examine if these subgroups had prognostic significance for patients with gastrointestinal DLBCL; (3) to assess the therapeutic outcomes of the different immunophenotype subgroups.

## **Methods**

### **Patients**

Between 1998 and 2010, there were 156 patients studied at Shanghai Renji Hospital with a histopathological diagnosis of primary gastrointestinal DLBCL. All the patients were enrolled in the course of the study that was approved by the local ethic committee. Written informed consent was obtained. Patient follow-up occurred through May 8, 2011. There were 151 patients followed up (96.8 %) and 5 patients lost. The median age was 63 years (range 15–81); and there were 91 men (60.3 %) and 60 women (39.7 %). All cases fulfilled the clinical criteria of primary gastrointestinal lymphoma presented by Dawson [2]. Among the 151 cases, 66.2 % (100/151) involved the stomach (48, gastric antrum; 35, gastric corpora; 10, gastric fundus; 7, multifocal) and 33.8 % (51/151) involved the intestine (21, ileocecal junction; 15, small intestine; 9, colon; 6, multifocal).

All 151 patients were pathologically diagnosed by surgery or endoscopic biopsy. Forty patients underwent radical or palliative resection only, 28 were treated with chemotherapy only, and 83 with surgery plus chemotherapy. Among the 111 patients treated with chemotherapy, 81 patients received CHOP-like therapy (CTX 600 mg/m2, d1; ADM 50 mg/m2 or EADM 70 mg/m2, d1; VCR 2 mg, d1; PRED 80 mg/m2, d1-5) and 30 patients received rituximab plus CHOP (R-CHOP) therapy. The median follow-up time was 30 months (range, 3 to 112 months).

### **Immunohistochemical investigation**

Immunohistochemical staining was performed with a two-step EnVision method. All tissue biopsies were fixed routinely in 10 % buffered formalin, embedded in paraffin, and cut into 4-μm sections. Following deparaffinization, heat-induced antigen retrieval techniques were used. Endogenous peroxidase activity was then blocked with 0.5 % H2O2. After being washed in Trisbuffered saline (TBS), the sections were stained with antibodies to CD10 (RTU-CD10-270-qh, Novocastra), BCL-6 (RTU-BCL-6-564-qh, Novocastra) and MUM1 (GM725902, DAKO). The reaction was carried out at room temperature overnight. After the sections were washed in TBS again, the secondary antibodies EnvisionTM (mouse) (K4001, DAKO) or EnvisionTM (rabbit) (K4002, DAKO) were dropped. The cell nucleus was restained with hematoxylin after Diaminobenzidine (DAB) showed color. Cases were considered positive if 30 % or more of the tumor cells were stained.

### **Immunohistochemical scoring**

The intensity of the staining as well as the percentage of positive cells was recorded. Staining intensity was scored from 0 to 3+; the intensity score was established as follows: 0, if absence of staining was observed; 1+, if >25 % of the tumor cells had weak staining; 2+, if tumor cells had moderate staining; and 3+ if tumor cells had strong staining. Tumors with 1+, 2+, and 3+ expression were interpreted as positive and tumors with no expression (0 score) were interpreted as negative.

### **Classification of DLBCL**

**Figure 1:**
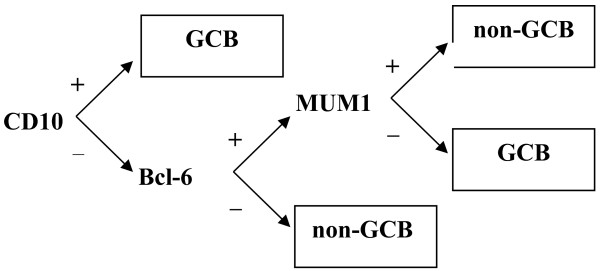
**Classification of gastrointestinal DLBCL.** According to CD10, Bcl-6 and MUM1 expression, 151 cases were classified into GCB and non-GCB types.

Using the Hans algorithm, according to CD10, BCL-6 and MUM1 expression, 151 cases were classified into two subtypes: GCB and non-GCB (Figure 1).

### **Statistical analysis**

The analysis was conducted with SPSS16.0 software. Characteristics of the two groups were compared using the χ2 test. The Kaplan-Meier method was used to estimate the survival distributions. The log-rank test was used to compare the survival distributions. All statistical tests were two-sided, and P < 0.05 was considered statistically significant.

## **Results**

At the time of analysis, 78 patients (51.7 %) were alive and 73 patients (48.3 %) had died. The overall 2, 3 and 5-year survival rates were 64.4 %, 56.2 % and 45.6 %, respectively. Expression of CD10 was observed in 22.5 % (34/151) of the patients, BCL-6 in 70.2 % (106/151), and MUM1 in 72.8 % (110/151). According to the results from immunohistochemical staining, 31.8 % (48/151) of the cases were classified as GCB subtype and 68.2 % (103/151) as non-GCB subtype.

The pathological parameters of the cases treated with surgery included tumor size, infiltrate depth and local lymph node metastasis. Statistical analysis showed that immunophenotype classification was not associated with tumor size and infiltrate depth. The mean diameter of tumors with two subtypes were 8.1±3.4 cm and 7.4±5.2 cm, respectively (T = −0.877, P > 0.05). And the tumors were localized within submucosa in 27.0 % (10/37) of GCB cases and 17.4 % (15/86) of non-GCB cases (χ2 = 1.468,P > 0.05) (Table 1). However, the significant association of subclassification and local lymph node metastasis was observed. There were 40.5 % (15/37) of GCB cases and 65.1 % (56/86) of non-GCB cases with local lymph node metastasis, respectively (χ2 = 6.403,P < 0.05) (Table 2).

**Table 1:** The infiltrate depth of tumors in GCB and non-GCB subtypes

	**infiltrate depth of tumors**		**χ2**	**P**
	**Mucosa and submucosa**	**Muscle layer and serosal**		
GCB	10(27.0 %)	27(73.0 %)	1.468	0.232
Non-GCB	15(17.4 %)	71(82.6 %)		

**Table 2:** Local lymph nodes metastasis in GCB and non-GCB subtypes

	**local lymph nodes metastasis**		**χ**^**2**^	**P**
	**(+)**	**(−)**		
GCB	15(40.5 %)	22(59.5 %)	6.403	0.017
Non-GCB	56(65.1 %)	30(34.9 %)		

**Figure 2:**
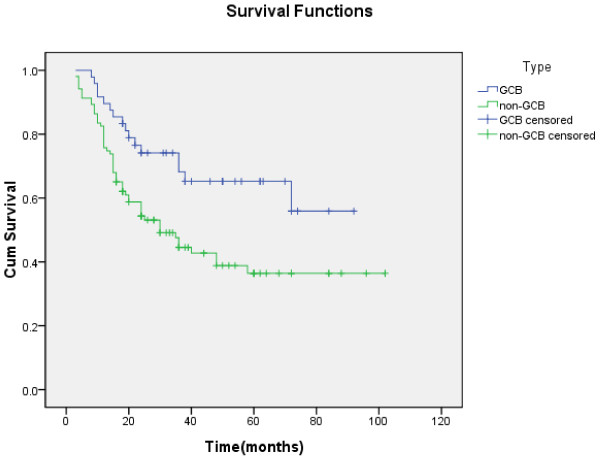
**Kaplan-Meier curve of OS for patients of GCB (blue) and non-GCB (green).** Patients of GCB lived longer than those of non-GCB (P < 0.05).

Univariate analysis of the expression of each protein and its relationship to survival are shown in Table 3. The 5-year survival rate was not significantly different between patients with CD10 positive and negative, BCL-6 positive and negative, or MUM1 positive and negative. However, survival analysis demonstrated that patients of GCB subtype had a significantly better survival rate compared with patients of non-GCB subtype (5-year survival rate, 65.2 % vs 36.4 %, χ2 = 7.371,P < 0.05) (Figure 2).

**Table 3:** Immunohistochemical stain results and their effect on survival by univariate analysis

		**No. (%)**	**5-year survival rate (%)**	**χ**^**2**^	**P**
CD10	Negative	77.5 % (117/151)	40.2 %	3.501	0.061
	Positive	22.5 % (34/151)	62.4 %		
Bcl-6	Negative	29.8 % (45/151)	36.1 %	1.328	0.249
	Positive	70.2 % (106/151)	49.5 %		
MUM1	Negative	27.2 % (41/151)	45.8 %	1.713	0.191
	Positive	72.8 % (110/151)	42.6 %		

**Figure 3:**
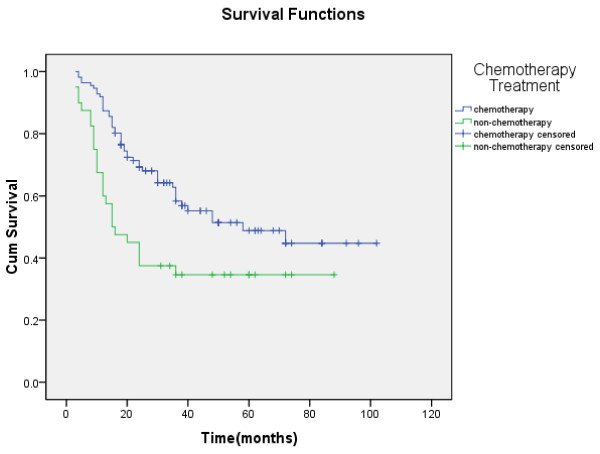
**Kaplan-Meier curve of OS for patients treated with chemotherapy (blue) and non-chemotherapy (green).** Patients treated with chemotherapy lived longer than those treated with non-chemotherapy (P < 0.05).

**Figure 4:**
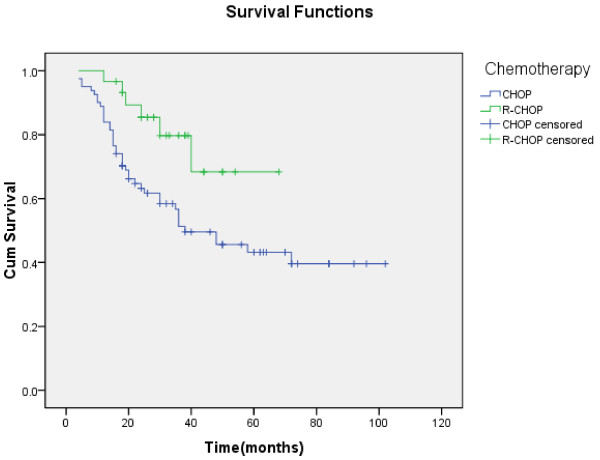
**Kaplan-Meier curve of OS for patients treated with CHOP (blue) and R-CHOP (green) postoperative chemotherapy.** Patients treated with R-CHOP lived longer than those treated with CHOP chemotherapy (P < 0.05).

In univariate analysis, chemotherapy was associated with improved survival. Patients treated with chemotherapy showed superior survival rates when compared to those treated with non-chemotherapy treatment (5-year survival rate, 48.8 % vs 34.6 %, χ2 = 8.440, P < 0.05) (Figure 3). The survival rate was not significantly different between patients treated with surgical and non-surgical treatment (5-year survival rate, 60.5 % vs 54.8 %, χ2 = 0.036, P > 0.05). Among the 111 patients who received chemotherapy, the survival rate for patients in the R-CHOP group was significantly longer than those in the CHOP group (5-year survival rate, 68.4 % vs 43.2 %, χ2 = 5.430, P < 0.05) (Figure 4).

We then compared the survival rate of patients treated with CHOP or R-CHOP therapy in different immunophenotype groups. In the GCB group, the 5 patients treated with R-CHOP therapy all survived. But there was no significant difference in the survival rate between the patients who received R-CHOP and CHOP therapy (χ2 = 1.981, P > 0.05). In the non-GCB group, the survival rate of patients treated with R-CHOP therapy was significantly longer than those with CHOP therapy (5-year survival rate, 62.8 % vs 30.8 %, χ2 = 6.140, P < 0.05).

## **Discussion**

DLBCL is the most common type of all non-Hodgkin’s lymphomas (NHLs) and accounts for 30 % to 40 % of new diagnoses. DLBCL is defined as diffuse proliferations of large neoplastic mature B cells, and it is, however, recognized that this definition comprises a group of morphologically, immunohistochemically, and clinically heterogeneous tumors rather than one single entity.

According to the literature, 27-48 % cases of non-Hodkin’s lymphoma are extranodal lymphoma. The gastrointestinal tract is the most common extranodal site of lymphoma, and the most common type of gastrointestinal lymphoma is DLBCL [3]. Yet there is hardly any research specifically focused on gastrointestinal DLBCL.

The original criteria for primary gastrointestinal DLBCL are: (1) pathologically confirmed diagnosis of DLBCL, according to the WHO classification; (2) clinically confirmed diagnosis of primary gastrointestinal lymphoma. In 1961, Dawson et al. proposed clinical criteria for distinguishing primary gastrointestinal lymphoma from secondary involvement. These criteria include: a) absence of peripheral lymphadenopathy at the time of presentation, b) lack of enlarged mediastinal lymphnodes, c) a normal total WBC and differential, d) predominance of the gastrointestinal lesion at the time of laparotomy with only the lymph nodes in the immediate vicinity being obviously affected, e) the liver and spleen not showing any lymphomatous involvement [2]. The 151 cases in our study fulfilled all these criteria.

During the past few years, several studies with cDNA microarrays have shown that DLBCL can be divided into three types according to the different stages of tumorigenic B-cell [4–9]. One of the three types, GCB DLBCL, expresses genes characteristic of normal GCB cells (expressing CD10, BCL-6, LMO-2, A-myb, BCL-7A, etc) and is associated with a good outcome after multiagent chemotherapy. Another type, activated B cell-like (ABC) DLBCL expresses genes characteristic of activated blood B cells (expressing IRF4, FLIP BCL-2, etc) and is associated with a poor clinical outcome. The third type, DLBCL co-express GCB and ABC DLBCL, is referred to as type 3. The type 3 group is heterogeneous and not well defined, but has a poor outcome similar to the ABC group. The study by Hans et al [1] showed that the 5-year overall survival rate for the GCB group is more than 70 %, which is obviously higher than the ABC and type 3 groups. The 5-year overall survival rate for ABC and type 3 is about 30 %.

Because gene expression technology is not currently available for routine clinical use and the technology requires fresh tissue with an adequate amount of RNA, the clinical application of classification of DLBCL by cDNA microarrays is restricted. In 2004, Hans et al found that tissue microarrays (TMA) could be used in the classification of DLBCL. The GCB and non-GCB subtypes can be accurately predicted according to the expression pattern of CD10, BCL-6 and MUM1. Compared with the cDNA microarray, the immunostain panel reproduced the gene expression results in 71 % of GCB and 88 % of non-GCB cases and predicted for survival in a similar manner. Results suggested that DLBCL can be divided into GCB and non-GCB subtypes by immunohistochemistry and that the non-GCB subtype probably included ABC type and type 3 [1]. Recent studies have demonstrated that the immunophenotype is an important independent prognostic factor [10,11].

In the current study, we aimed to test the expression of CD10, BCL-6 and MUM1 by immunohistochemical staining. CD10 is a membrane-associated, neutral endopeptidase, which is expressed in a variety of human tissue, but has a restricted expression in the germinal center cells of reactive lymphoid tissue [12]. BCL-6 is a zinc-finger protein that acts as a transcriptional repressor and is expressed in germinal center B cells and a subset of CD4+ T cells [13–16]. CD-10 and BCL-6, regarded as the important markers in diagnosing GCB DLBCL, both express in normal GCB cells. Furthermore, many studies have suggested that expression of CD10 and BCL-6 in DLBCL predicts better overall survival rates. MUM1 is a lymphoid-specific member of the interferon regulatory factor family of transcription factors [17]. MUM1 is normally expressed in plasma cells and a minor subset of germinal center cells. Expression of MUM1 may denote the final step of germinal center B-cell differentiation with subsequent B-cell maturation toward plasma cells [18]. It appears that MUM1 has the potential to be a marker of the non-GCB phenotype.

We found that, of 151 cases of gastrointestinal DLBCL, 48 (31.8 %) were classified as GCB and 103 cases (68.2 %) as non-GCB. The study on the relationship between immunophenotype classification and pathological parameters indicated that patients of non-GCB subtype were more likely to suffer from local lymph node metastasis than patients of GCB subtype, while the two subtypes did nothing with tumor size and infiltrate depth. Our study also showed that the survival rate of patients in the GCB group was obviously longer than that of patients in non-GCB group. These results were similar to those of Hans’s study which focused on both nodal and extranodal DLBCL [1]. The results above indicate that the immunophenotype classification could be used to predict the prognosis of the primary gastrointestinal DLBCL patients.

During the past 10 years, the treatment of PGI-DLBCL was still controversial. In a current study, chemotherapy was associated with improved survival rates while surgical treatment did not significantly affect it. Most previous studies have suggested that chemotherapy or surgery plus postoperative chemotherapy significantly improved survival of patients with gastrointestinal lymphoma. Our finding was similar to them. But the patients in most studies (including ours) were not randomized to treatment as part of a clinical trial, so there were some possible confounding factors. For example, older or sicker patients were not candidates for chemotherapy. We consider that randomized control trial should be performed to assess the therapeutic outcomes.

Several recent studies showed that R-CHOP significantly improves the clinical outcome of patients with DLBCL [19-22]. In a retrospective study by Nyman et al[23], they found that the additional benefit of R-CHOP extended only to patients with non-GCB DLBCL and not those with GCB DLBCL. In addition, Nyman et al did not find a difference in survival between GCB and non-GCB subtypes in the post-rituximab era, which implies that the addition of rituximab eliminates the prognostic significance of the classification of DLBCL. In a current study, the survival rate of patients treated with R-CHOP chemotherapy was significantly longer than that to patients treated with CHOP. And in the non-GCB group, the survival rate of patients treated with R-CHOP was significantly longer than those with CHOP. In the GCB group, R-CHOP chemotherapy also seemed to be an advantage (5 patients receiving R-CHOP chemotherapy survived). But it was not found to be statistically significant between patients receiving R-CHOP and CHOP therapy. These results implied that the immunophenotype was useful in predicting prognosis as well as selecting the most economical, effective and reasonable chemotherapy regime. R-CHOP chemotherapy is an advisable choice to patients of non-GCB subtype, which can overcome the adverse effects of this subtype. And if the patient’s economic conditions permit, R-CHOP is also the first choice to patients of GCB subtype who can also be treated with CHOP alone. However, because our study is retrospective and the patient population is small (especially the cases treated with R-CHOP therapy in GCB group may be not enough), further studies need to be performed on a larger patient population with primary gastrointestinal DLBCL to confirm the results.

## **Conclusions**

From our results we can conclude that the immunophenotype classification of gastrointestinal DLBCL, which is closely related to local lymph node metastasis, is found to have prognostic significance. Immunophenotype classification is also useful in selecting the chemotherapy protocol.

## **Abbreviations**

DLBCL, Diffuse large B-cell lymphoma; GCB, Germinal center B cell-like; TMA, tissue microarrays.

## **Competing interests**

The authors declare that they have no competing interests.

## **Authors’ contributions**

Zizhen Zhang did the main part of the data collection, performed the statistical analysis and drafted the manuscript. Yanying Shen coordinated and performed the work done in the laboratory. Danping Shen contributed to the data collection and helped to draft the manuscript. Xingzhi Ni as principal supervisor helped to design the study and reviewed the draft of the manuscript. All authors read and approved the final manuscript.

## Pre-publication history

The pre-publication history for this paper can be accessed here:

http://www.biomedcentral.com/1471-230X/12/77/prepub
